# A Brief Assessment of Body Image Perception: Norm Values and Factorial Structure of the Short Version of the FKB-20

**DOI:** 10.3389/fpsyg.2020.579783

**Published:** 2020-12-01

**Authors:** Ileana Schmalbach, Bjarne Schmalbach, Markus Zenger, Hendrik Berth, Cornelia Albani, Katja Petrowski, Elmar Brähler

**Affiliations:** ^1^Technische Universität Dresden, Carl Gustav Carus Faculty of Medicine, Division of Psychological and Social Medicine and Developmental Neurosciences, Research Group Applied Medical Psychology and Medical Sociology, Dresden, Germany; ^2^Department of Medical Psychology and Medical Sociology, Johannes-Gutenberg University Mainz, Mainz, Germany; ^3^Department of Applied Human Studies, University of Applied Sciences Magdeburg and Stendal, Stendal, Germany; ^4^Integrated Research and Treatment Center (IFB) Adiposity Diseases – Behavioral Medicine, Medical Psychology and Medical Sociology, University of Leipzig Medical Center, Leipzig, Germany; ^5^Department of Psychotherapy and Psychosomatic Medicine, University Hospital Leipzig, Leipzig, Germany; ^6^Department of Psychosomatic Medicine and Psychotherapy, University Medical Centre, Johannes Gutenberg University Mainz, Mainz, Germany

**Keywords:** body image, body dysmorphia, eating disorders, ideal body image, scale construction, Body Image Questionnaire-6

## Abstract

The Body Image Questionnaire-20 (FKB-20) is one of the most applied self-report measures in the context of body image assessment in German-speaking regions. A version of the FKB-20 capturing an ideal concept of body image is also available. A special property of the scale is its high sensitivity for individuals suffering from anorexia nervosa. The present research provided a short version of this scale (for both variants) and examined its validity in a representative sample (*N* = 2,347) of the German population. We utilized factor analysis methods to identify the optimal short scale of the measure, finding excellent model fit and reliability for a two-factor model (FKB-6) for both real and ideal body image. Both versions of the FKB-6 can be considered invariant across sex and age groups. Good reliability indices were shown for both versions of the FKB-6. The reliability indices were similar to those mentioned in previous studies. Our study also revealed, that large discrepancies between the real and an ideal body image are correlated with somatic and body dysmorphic symptoms. Finally, we provided norm values for comparisons of individual scores with the general population. The FKB-6 is a valid and a reliable measure that economizes assessments by clinicians and researchers.

## Introduction

The diagnosis of body image perturbances in eating (e.g., anorexia nervosa, bulimia nervosa), and in other psychiatric disorders or illnesses involving physical changes and disturbances associated with body dissatisfaction (e.g., sexual dysfunction, conversion disorders, transsexualisms, cancer) is part of the everyday clinical practice (Albani et al., 2006a; Peterson et al., 2017; Steinfeld et al., 2017; Groot, 2020). In this light, treatment and recovery are major goals during therapy. To that end, the application of validated instruments to measure body image components that allow the mapping of inter-individual differences and intra-individual development processes is indispensable (Cash, 2017).

Body image is understood as a multifaceted construct comprising a perceptual and an attitudinal component. The former indicates the accuracy of an individuals’ judgment of size, shape and form relative to their actual body proportions, while the latter reflects the affective dimension of the construct (Cash and Smolak, 2011; Thompson and Schaefer, 2019). The attitudinal component conveys at least two dimensions. The first involves body image appraisals and feelings toward one’s appearance (evaluative-affective). The second, emphasizes cognitive-behavioral variables on one’s appearance, e.g., thoughts, concerns and internalized ideals (Cash, 2012). Cash (2012) refer to this dimension as *investment*. Body image experiences vary across life span and situational contexts (Tiggemann, 2004; Quittkat et al., 2019). However, there are other factors that directly affect body image, such as gender, cultural and social norms, childhood experiences, as well as biological factors—among others (Daig et al., 2006; Nichols et al., 2018; Saiphoo and Vahedi, 2019). In sum, an individual’s judgment on the perception of their body is strongly influenced by cognitive and affective variables, as well biological factors and social norms.

The construct of body image has its roots in clinical settings and has been applied in studies related to neurological disorders, eating or weight disorders and body dysmorphophobia (Slade, 1994; Di Cara et al., 2019; McLean and Paxton, 2019). Accordingly, it has been shown that body image is associated with psychosocial functioning (Cash and Fleming, 2002; Fatt et al., 2020). Many studies show that a negative body image is correlated with low self-esteem, depression, poor physical well-being, low quality of life and hypochondriasis (Cash and Smolak, 2011; Wilson et al., 2013; França et al., 2017; Becker et al., 2019), while a positive attitude is linked to high-levels of well-being, self-esteem, weight stability, self-care and physical activities as well as proactive coping behaviors (Cash and Fleming, 2002; Zanon et al., 2016; Swami et al., 2018; Sabiston et al., 2019).

Even if there are different methods for body image assessment (Löwe and Clement, 1996; Joraschky et al., 2018; Thompson and Schaefer, 2019) difficulties in operationalizing body image have been continuously claimed. For example, the insufficient evaluation of psychometric properties has been constantly pointed out (Thompson, 2004; Kling et al., 2019; Thompson and Schaefer, 2019). In the past, some researchers (Daig et al., 2006) stated that body image is not measured broadly enough, since affective, cognitive and behavioral aspects were overlooked. Such variables are essential when analyzing body image discrepancies (Daig et al., 2006; Joraschky et al., 2018), as explained by Higgins (1987) in his “self-discrepancy theory.” The author described three dimensions of the self (actual, ideal, ought selves) and two perspectives: the own and the significant others’. It is suggested that individuals strive for the best possible fit between the current self-concept and internalized ideals. Discrepancies between the ideal and the current body image are related to negative appraisals, body dissatisfaction and body dysmorphic symptoms (Daig et al., 2006; Glauert et al., 2009; Vashi, 2016).

Body dissatisfaction has been increasingly observed (Halliwell et al., 2011; Wang et al., 2019). An influential contribution of this phenomena might be anchored on the discrepancy between beauty ideals (e.g., thinness for women and muscular body for males; Mills et al., 2017; Nagar and Virk, 2017) and average body sizes of women and men. Since body image dissatisfaction might have pervasive consequences for quality of life (Cash and Fleming, 2002; Mond et al., 2013) and it is also associated with a variety of psychiatric conditions, its assessment has exponentially increased (Cash, 2002; Kling et al., 2019; Thompson and Schaefer, 2019; Wang et al., 2019). Diagnosis and treatment of body image disturbances are key for psychological functioning and well-being. While there is a variety of body image measures available for English-speaking regions, the scarcity of validated scales (Kling et al., 2019) suitable for German-speaking regions has been pointed out (Löwe and Clement, 1996; Thompson, 2004; Albani et al., 2006a). From this perspective, the main purpose of the study is to provide a valid and economic tool suitable for daily clinical practice in German-speaking populations aiming to target body image concerns, by means of a short version of the FKB-20 (Fragebogen Körperbild-20 = Body Image Questionnaire-20; Löwe and Clement, 1996).

The FKB-20 is considered to be one of the most applied self-report measures (in German-speaking areas) in its field. It was developed to diagnose body image disorders in clinical practice and measure body image-related aspects (Löwe and Clement, 1996). A peculiarity of the scale is the focus on measuring a stable concept of one’s body image, rather than a current state. The FKB-20 integrates cognitive, affective and evaluative aspects of body image comprised in two subscales *Rejecting Body Image* (RBI; evaluations of an individual’s body image regarding appearance and well-being) and *Vital Body Dynamics* (VBD; energetic and movement related aspects and activities). This measure has been applied in numerous studies and clinical settings, showing satisfactory psychometric properties and especially a high sensitivity to change and detect eating disorders, e.g., anorexia nervosa (Löwe and Clement, 1996; Albani et al., 2006a; Mölbert et al., 2018). It was also applied to specify body dysmorphia disorder (Sack et al., 2002; Schieber and Martin, 2016) and measure the effects of different group therapy interventions in patients with multiple sclerosis (Tesar et al., 2003; Veleva et al., 2018), cancer (Grübel, 2003; Esser et al., 2018), a heart attack (Löwe et al., 2002) and obesity (Hotter et al., 2003; Ziser et al., 2019). Besides that, it was revealed that patients with a mental illness or pronounced psychological distress reported higher values on the RBI subscale and lower values on the VBD subscale (Löwe et al., 2003). Another uniqueness of the FKB-20 is the availability of a version that measures one’s concept of an ideal body image. Such was validated by Daig et al. (2006) by reformulating the original items into the third person. In their research, they reported significant differences between the means of the two versions and correlations to body dysmorphic symptoms. High discrepancies were observed in young women in terms of RBI. The higher the scores on the VBD (in both versions of the scale), the lower the body dysmorphic symptoms were. This also holds true for the RBI, but in the opposite direction: the higher the RBI-scores (in both versions), the stronger the body dysmorphic symptoms.

Considering the growing demand for scientifically sound measures in the field of body image assessment in German-speaking regions and the value of such in clinical and in research settings, a short version appears convenient. Especially for patients with a background of mental illness, older participants, or for large-scale health surveys, an economical measure seems desirable. Therefore, the main aim of the present study is to develop a short scale for each version of the FKB-20 and evaluate their psychometric properties. A further aim is to provide insight of how body image perception affects relevant psychological variables. For this purpose, we explored discrepancies between the real (FKB-6_*r*__*eal*_) and the ideal body image (FKB-6_*i*__*deal*_) and their relation to: physical and subjective well-being, quality of life, somatoform and body dysmorphic symptoms.

To provide evidence of the validity of the *FKB-20*, correlations with the following measures will be evaluated: *Questionnaire for* assessing *subjective physical well-being (FEW), EURO-HIS-QOL-8 (Health Related Index of Quality of Life), The World Health Organization-Five Well-Being Index (WHO-5), Screening for Somatoform Disorders (SOMS)* and *Body dysmorphic symptoms inventory (BDSI)*. Physical well-being (FEW), quality of life (HIS-QOL-8) and subjective well-being (WHO-5) are expected to be positively associated to VBD, but weakly or negatively related to RBI (FKB-6_*r*__*eal, ideal*_; Cash and Fleming, 2002; Sinclair and Myers, 2004; Williams et al., 2004; Rief et al., 2006). In addition, somatoform (SOMS) and body dysmorphic symptoms (BDSI) are expected to positively correlate with RBI, but negatively with VBD (FKB-6_*r*__*eal, ideal*_; McCaulay et al., 1988; Carlson, 2004; Tiggemann, 2004; Sarwer et al., 2005; Cash and Smolak, 2011). Moreover, we hypothesized that discrepancies between the real and the ideal body image (in both dimensions: VBD, RBI) will be positively correlated with somatoform and body dysmorphic symptoms and negatively with physical and subjective well-being as well as with quality of life (Daig et al., 2006; Glauert et al., 2009).

## Materials and Methods

### Participants and Procedure

The data in the present study were collected on behalf of the local university, supported by a demographic research institute (USUMA, Berlin) as part of a population-based representative survey using a multistage sampling. The participants were selected by a random-route method and visited at home by trained and experienced interviewers from the research institute USUMA, Berlin. From all the household members the target participant at home was also randomly selected (Kish-Selection-Grid). Inclusion criteria required subjects to be at least 14 years of age in addition to sufficient command of the German language. As part of the interviews, participants filled in self-report questionnaires (participation was voluntarily). Each respondent received a signed privacy policy form. Parents or legal guardians signed a consent form approving the participating of underaged subjects. The response rate of the survey was 62.3%. Drop outs were due to dismissal of participation by the household (14%) or by the target subject (9.4%), failed attempts to contact the selected household (9%) and the target subject (3.6%). Additionally, some interviews were invalid (*n* = 39; items missing values). The final sample consists a total of *N* = 2,429 participants between 14 and 99 years of age (50.35; *SD* = 17.44), with 52.9% of the subjects being female. The methods of data collection were in accordance with the Helsinki Declaration of 1975 (as revised in 1983). Further details on the sociodemographic composition of the sample are described in Table 1. The sample is representative in terms of respondent age and sex when compared to data from the Federal Statistical Office of Germany (2019). There were no significant deviations as per χ^2^ ≤ 4.00, *p* ≥ 0.550, *V* ≤ 0.063. However, - at least descriptively—we acknowledge a underrepresentation of individuals of age 70 or older.

**TABLE 1:** Sample description with group comparisons for the FKB-6.

	***n***	**%**	**% in pop.**	**FBK-6, *M*(*SD*)**			

				**Real body image-version**		**Ideal body image-version**	
				**RBI**	**VBD**	**RBI**	**VBD**
Sex				*F*(1, 2,366) = 27.061, *p* = 0.001, η^2^*_*p*_* = 0.011	*F*(1, 2,366) = 28.090, *p* = 0.001, η^2^*_*p*_* = 0.012	*F*(1, 2,366) = 12.118, *p* = 0.001, η^2^*_*p*_* = 0.005	*F*(1, 2,366) = 17.082, *p* = 0.001, η^2^*_*p*_* = 0.007
Female	1,186	50.4	50.7	1.840 (0.841)	3.611 (0.907)	1.911 (0.837)	3.746 (0.866)
Male	1,167	49.6	49.3	1.663 (0.810)	3.812 (0.937)	1.791 (0.845)	3.897 (0.911)
Age (*M* = 46.16; *SD* = 17.98)				*F*(5, 2,366) = 2.277, *p* = 0.045, η^2^*_*p*_* = 0.005	*F*(5, 2,366) = 111.162, *p* =.001, η^2^*_*p*_* = 0.191	*F*(5, 2,366) = 1.531, *p* = 0.177, η^2^*_*p*_* = 0.003	*F*(5, 2,366) = 73.949, *p* = 0.001, η^2^*_*p*_* = 0.135
18–29	491	20.9	20.4	1.690 (0.848)	4.189 (0.789)	1.852 (0.885)	4.156 (0.791)
30–39	442	18.8	14.4	1.678 (0.812)	4.021 (0.794)	1.804 (0.838)	4.081 (0.731)
40–49	417	17.7	14.8	1.811 (0.869)	3.838 (0.823)	1.833 (0.822)	3.981 (0.782)
50–59	337	14.3	18.5	1.812 (0.862)	3.657 (0.888)	1.914 (0.925)	3.981 (0.782)
60–69	416	17.7	13.9	1.802 (0.799)	3.357 (0.831)	1.917 (0.799)	3.787 (.861)
≥ 70	251	10.7	18.0	1.747 (0.777)	2.932 (0.913)	1.791 (0.769)	3.154 (1.001)
Education				*F*(3, 2,366) = 12.458, *p* = 0.001, η^2^*_*p*_* = 0.016	*F*(3, 2,366) = 63.914, *p* = 0.001, η^2^*_*p*_* = 0.075	*F*(3, 2,366) = 0.813, *p* = 0.487, η^2^*_*p*_* = 0.001	*F*(3, 2,366) = 36.558, *p* = 0.001, η^2^*_*p*_* = 0.044
≤ 9 years	1,060	45.0		1.845 (0.858)	3.438 (0.942)	1.882 (0.849)	3.621 (0.911)
10 years	793	33.7		1.740 (0.841)	3.906 (0.841)	1.827 (0.830)	3.954 (0.838)
≥ 11 years	412	17.5		1.557 (0.686)	3.955 (0.869)	1.828 (0.845)	4.018 (0.835)
School students	88	3.7		1.700 (0.870)	4.231 (0.766)	1.841 (0.895)	4.220 (0.824)
Family				*F*(5, 2,366) = 63.914, *p* = 0.001, η^2^*_*p*_* = 0.191	*F*(5, 2,366) = 59.897, *p* = 0.001, η^2^*_*p*_* = 0.113	*F*(5, 2,366) = 3.158, *p* = 0.008, η^2^*_*p*_* = 0.007	*F*(5, 2,366) = 39.785, *p* = 0.001, η^2^*_*p*_* = 0.078
Married	1,420	60.4		1.719 (0.812)	3.727 (0.876)	1.828 (0.835)	3.843 (0.855)
Committed relationship	103	4.4		1.670 (0.759)	4.010 (0.889)	1.770 (0.787)	3.974 (0.721)
Single	464	19.7		1.752 (0.877)	4.058 (0.846)	1.881 (0.877)	4.037 (0.858)
Separated	22	1.6		1.892 (1.000)	3.535 (0.857)	2.107 (0.902)	3.666 (0.551)
Divorced	164	7.0		1.825 (0.833)	3.672 (0.887)	1.765 (0.808)	3.946 (0.846)
Widowed	179	7.6		1.875 (0.823)	2.945 (0.899)	1.997 (0.843)	3.170 (0.935)
Employment				*F*(4, 2,366) = 3.263, *p* = 0.011, η^2^*_*p*_* = 0.005	*F*(4, 2,366) = 125.498, *p* = 0.001, η^2^*_*p*_* = 0.175	*F*(4, 2,366) = 0.739, *p* = 0.565, η^2^*_*p*_* = 0.001	*F*(4, 2,366) = 74.190, *p* = 0.001, η^2^*_*p*_* = 0.112
Working fulltime	886	37.7		1.723 (0.851)	4.024 (0.781)	1.825 (0.877)	4.047 (0.781)
Working part- time	228	9.7		1.742 (0.787)	3.821 (0.823)	1.842 (0.733)	3.975 (0.7003)
Unemployed	375	15.9		1.849 (0.885)	3.705 (0.880)	1.907 (0.895)	3.832 (0.857)
Retired	629	26.7		1.788 (0.809)	3.146 (0.917)	1.853 (0.802)	3.380 (0.954)
In training	234	10.0		1.608 (0.729)	4.207 (0.771)	1.891 (0.850)	4.177 (0.779)

*FBK-6 = Six-item version of the Body Image Questionnaire; % in pop = Distributions in the German general population, according to the Federal Statistical Office of Germany (2019).*

### Instruments

#### Body Image Questionnaire (FKB-20; Clement and Löwe, 1996; Albani et al., 2006a)

This scale measures cognitive, affective, and evaluative variables of an individual’s concept of body image and reflects relatively time-stable physical aspects, rather than current physical conditions. The questionnaire comprises two subscales: *Rejecting Body Image* (α = 0.80; e.g., *My body is often a burden to me, I am not happy with my body shape*) and *Vital Body Dynamics* (α = 0.90*;* e.g., *I am healthy, I am in top shape*) with 10 items each, ranging from 1 = *it does not apply* to 5 = *it applies*. The subscale scores are calculated by summing up the item values. Satisfactory psychometric values have been demonstrated in previous studies (e.g., Grübel, 2003; Tesar et al., 2003; Albani et al., 2006a; Daig et al., 2006). For the ideal body image version of the FKB-20 (Daig et al., 2006), the original items were rephrased into the third person. Participants were asked to picture a person of the same age and gender and describe their ideal physical sensations as well as an optimal body image (e.g., “He/She feels full of strength”). In the present study, the correlations between the real and ideal body image versions were high: VBD *r* = 0.73, RBI *r* = 0.62 and Cronbach’s Alpha values (for both versions) were satisfactory (VBD = α = 0.88, RBI = α = 0.86).

*EURO-HIS-QOL-8* (EURO-Health Related Index of Quality of Life-8; Brähler et al., 2007). This scale assesses the general quality of life regarding psychological, physical, social, and environmental variables. It comprises 8 items which refer to the short version of the two questionnaires WHO-QOL-100 (Power et al., 1999) and WHO-QOL-BREF (Skevington et al., 2004). Answers are to be rated on a five-point scale (1 = *very bad* to 5 = *very good*) based on the past 2 weeks. The total score is achieved by summing up the item scores. Extensive surveys with the questionnaire provide evidence of good psychometric properties (α = 0.80–0.92; Gunzelmann et al., 2006; Schmidt et al., 2006).

#### World Health Organization Well-Being Index-5 (WHO-5; Brähler et al., 2007)

This measure is a short version of the WHO-10 (Bech et al., 1996) and it has been translated into more than 30 languages. The WHO-5 measures subjective psychological well-being by means of five positively phrased items. Each item is scored from 5 = *all of the time* to 0 = *at no time*, referring to a period within the past 2 weeks. The raw score ranges from 0 = *absence of well-being* to 25 = *maximal well-being*. The scale has been validated and applied in different settings showing satisfactory psychometric properties (α = 0.89–0.95; Heun et al., 2001; De Wit et al., 2007; Krieger et al., 2014; Topp et al., 2015).

#### Questionnaire for Assessing Subjective Physical Well-Being (FEW; Kolip and Schmidt, 1999; Albani et al., 2006b)

The questionnaire is applied to measure habitual physical well-being (and not the absence of complaints) by means of four dimensions: resilience, vitality, enjoyment, and inner peace. Each scale has four exclusive positively formulated items rated from 0 = *does not apply at all* to 5 = *applies completely*. The scale values are calculated as mean values of the associated items. The total value is calculated as the mean value of the four scales. Psychometrics are satisfactory (α = 0.88–0.93). Norm values are available (Albani et al., 2006a).

#### Body Dysmorphic Symptoms Inventory (BDSI; Buhlmann et al., 2009)

The BDSI is a screening tool that measures the severity of symptoms related to body dysmorphia, such as excessive preoccupation with disliked body parts and suicidality. The questionnaire also captures how much time is employed in worries and concerns related to disliked body regions (0 = *I don’t think about it* to 4 = *over 8 h/day or more*), as well as the extent of ritualized, appearance-related actions (0 = *not at all* to 4 = *over 8 h/day or more*). Furthermore, it reflects the strain and impairment caused in daily tasks due to preoccupation with body concerns. The scale is a self-report instrument comprising 18 items. Questions two and three serve as auxiliary items to detect eating disorders and are not included in the total score. The score of the BDSI ranges from 0 to 64. Additionally, four items capture the criteria of a body dysmorphic disorder based on the DSM-IV (Rief et al., 2006). Psychometric properties are satisfactory (α = 0.88; Buhlmann et al., 2009).

#### Screening for Somatoform Disorders (SOMS; Rief et al., 1997)

The SOMS serves as a screening tool for the criteria of somatoform disorders according to ICD-10 and DSM-IV. It assesses 53 symptoms that had been present in the past 7 days. Each symptom is rated according to its degree of associated impairment 1 = *mild* to 4 = *very severe*. In the present study we focus on the severity index for somatization (SSI), which is computed by summing up the item scores. The psychometric properties of the scale have shown satisfactory values in previous studies (α = 0.88–0.92; Rief et al., 1997; Hessel et al., 2002; Rief and Hiller, 2003; Hiller et al., 2006). The SSI discriminated patients with somatoform disorders from those with other forms of mental disorders.

### Statistical Analyses

All analyses were conducted in *R*, using the packages *EFAutilities*, *ezCutoffs, lavaan, multilevel*, *psych*, *semTools*, and *stuart* (Rosseel, 2012; Bliese, 2016; Revelle, 2018; Schmalbach et al., 2019; Schultze, 2019; Zhang et al., 2019; Jorgensen et al., 2020). Initially, we randomly split our full sample (*N* = 2,347) into an exploratory (*n* = 1,147) and a confirmatory one (*n* = 1,200). This will allow for independent testing in the confirmatory sample of the model generated in the exploratory sample. To determine the number of substantial factors, we then conducted parallel analysis (Horn, 1965). This procedure compares the raw, empirical eigenvalues to eigenvalues of randomly generated covariance matrices with the same properties as the original data set—and their 99% confidence interval.

Next, we used several methods of item reduction and model generation. First, we conducted exploratory factor analysis using ordinary least squares extraction and oblique rotation. Second, we examined item descriptive statistics. We then discarded all items that either exhibited loadings smaller than 0.500, item-total correlations smaller than 0.500, or absolute skewness and excessive kurtosis values larger than 2 or 4, respectively (Hair et al., 2013; Kim, 2013), or several of the above. Third, we used *stuart* to further reduce the item pool and generate a shortened model—with three items per factor—for testing in the confirmatory sample. *Stuart* utilizes ant colony optimization to construct and test subsets of a scale and maximize model fit. In addition, we constrained the algorithm to prefer solutions that are strongly invariant across participant’s gender.

We then ran confirmatory factor analysis based on robust maximum likelihood estimation (Satorra and Bentler, 2001) to test model fit in the confirmatory sample. To evaluate model fit, we inspected χ^2^ and the following model fit indices, comparing them to the commonly employed cut-off values: The Comparative Fit Index (*CFI*), the Tucker-Lewis-Index (*TLI*), the Root Mean Square Error of Approximation (*RMSEA*), and the Standardized Root Mean Square Residual (*SRMR*). *CFI* and *TLI* should be larger than 0.950, and *RMSEA* and *SRMR* should be smaller than 0.06 to evince good fit (Hu and Bentler, 1999; Schermelleh-Engel et al., 2003). To supplement these generic cut-off values, we calculated simulated cut-offs using *ezCutoffs* (Schmalbach et al., 2019). Using 1,000 simulated data sets based on the same number of observations and the same population model, ideal fit index cut-off values are determined empirically. Furthermore, it should be noted that we the utilized robust formulas for the estimation of *CFI*, *TLI*, and *RMSEA* (Broesseau-liard et al., 2012; Brosseau-Liard and Savalei, 2014). For the analysis of measurement invariance, we utilized the common procedure of comparing increasingly restrictive models (configural, metric, scalar, strict; Milfont and Fischer, 2010). If the differences in scaled χ^2^, robust *CFI* and robust *RMSEA* were non-significant and smaller than or equal to 0.01 for *CFI* and 0.015 for *RMSEA*, respectively, we judged it as evidence for measurement invariance (Chen, 2007). Finally, we used ω as a measure of factor score reliability (Dunn et al., 2014).

## Results

### Item Reduction

The results of the EFA and the item descriptive analyses are reported in Tables 2, 3a, b. Parallel analysis revealed clear evidence for a two-factorial structure, which was mirrored in the EFA results: Items 5, 13, and 19 exhibited loadings smaller than 0.500, crossloadings, or both. In addition, corrected item-total correlations exceeded 0.500 for all items except Items 5, 13, and 19. We supplemented these analyses by applying an analysis based on item response theory and investigated item discrimination parameters using a graded response model in the *mirt* package (Chalmers, 2012): Similar to the results from the EFA, Items 5, 13, and 19 evinced the lowest discrimination for the Rejecting Body Image scale. According to Baker (2001), Item 5 had low discrimination (*a*_5_ = 0.51), and Items 13 and 19 had moderate discrimination (*a*_13_ = 1.13, *a*_19_ = 1.28). All other items had high or very high discrimination. For the Vital Body Dynamic scale, only Item 20 had a discrimination parameter which was not at least high (*a*_20_ = 1.12).

**TABLE 2:** Results of the parallel analysis.

	**Raw eigenvalues**	**99th percentile**
1	7.08	1.29
2	3.34	1.24
3	0.88	1.20
4	0.77	1.17

**TABLE 3a:** Item descriptive statistics and factor loadings in confirmatory factor analysis real body image—version.

						**CFA**	
	***M***	***SD***	**γ1**	**γ2**	***r*_*it*_**	**VBD**	**RBI**
Item 3^a^	3.919	1.044	−0.978	0.535	0.750	0.806	
Item 7^a^	3.647	0.972	−0.577	−0.014	0.803	0.865	
Item 14^a^	3.556	1.071	−0.588	−0.192	0.810	0.848	
Item 6^b^	1.588	0.950	1.667	2.112	0.617		0.797
Item 8^b^	2.041	1.127	0.818	−0.339	0.480		0.540
Item 10^b^	1.637	0.968	1.571	1.887	0.665		0.827
FBK-6 RBI	1.755	0.831	1.2212	1.089			
FBK-6 VBD	3.707	0.927	−0.698	0.039			

*γ1, skewness; γ2, kurtosis; r_*it*_, corrected item-total correlation; RBI, Rejecting Body Image; VBD, Vital Body Dynamic. ^*a*^Part of the VBD subscale according to the manual. ^*b*^Part of the RBI subscale according to the manual.*

**TABLE 3b:** Item descriptive statistics and factor loadings in confirmatory factor analysis—ideal body image version.

						**CFA**	
	***M***	***SD***	**γ1**	**γ2**	***r*_*it*_**	**VBD**	**RBI**
Item 3^*a*^	3.961	0.997	−0.927	0.501	0.701	0.807	
Item 7^*a*^	3.773	0.987	−0.682	0.106	0.748	0.806	
Item 14^*a*^	3.721	1.051	−0.677	−0.027	0.754	0.823	
Item 6^*b*^	1.726	0.983	1.286	0.863	0.572		0.763
Item 8^*b*^	2.102	1.119	0.764	−0.290	0.538		0.659
Item 10^*b*^	1.731	0.990	1.324	1.086	0.600		0.729
FBK-6 RBI	1.853	0.843	0.891	0.115			
FBK-6 VBD	3.818	0.891	−0.675	0.077			

*γ1, skewness; γ2, kurtosis; r_*it*_, corrected item-total correlation; RBI, Rejecting Body Image; VBD, Vital Body Dynamic. ^*a*^Part of the VBD subscale according to the manual; ^*b*^Part of the RBI subscale according to the manual.*

By investigating skewness and kurtosis, we were able to eliminate Items 10 and 11 as a highly non-normally distributed items, with the vast majority of participants choosing the lowest response option. After eliminating these five items, we used the remaining 16 items as input for model generation in *stuart*. Among the possible 1,200 combinations, the algorithm selected those items marked in Table 2. In the exploratory sample, this solution evinced excellent fit, χ^2^(24) = 51.850, *p* < 0.001, *CFI* = 0.992, *TLI* = 0.989, *RMSEA* (90% *CI*) = 0.044 (0.028; 0.061), *SRMR* = 0.033. The item inter-correlations exceed 0.300 for all scales, and are, thus, satisfactory (Nunally and Bernstein, 1994).

### Confirmatory Analyses

We then tested this configuration in the confirmatory subsample, and were able to affirm the very good fit, χ^2^(8) = 20.488, *p* = 0.009, *CFI* = 0.995, *TLI* = 0.991, *RMSEA* (90% *CI*) = 0.040 (0.019; 0.062), *SRMR* = 0.021. As fixed rules of thumb for model fit evaluation have been criticized again and again (cf. Nye and Drasgow, 2011; McNeish et al., 2018), we utilized the simulation-based approach implemented in the R package *ezCutoffs* (Schmalbach et al., 2019) to determine model-specific cut-off values: χ^2^ = 19.998, *CFI* = 0.993, *TLI* = 0.987, *RMSEA* = 0.036, and *SRMR* = 0.024. Three of the five empirical indices were acceptable according to these cut-offs. Standardized factor loadings were greater than or equal to λ = 0.645 for all six indicators, and the latent variables correlated at *r* = −0.445. Reliability for both subscales was good, ω_*VBD*_ = 0.878 and ω_*RBI*_ = 0.757—particularly considering the brevity of the scales (6 items in total).

#### Ideal Body Image Version

A confirmatory analysis was also computed for the short form of the ideal version of the FKB-20. Results show a very good fit, χ^2^(8) = 18.438, *p* = 0.018, *CFI* = 0.994, *TLI* = 0.989, *RMSEA* (90% *CI*) = 0.041 (0.016; 0.065), *SRMR* = 0.021. Here four of the five fit measures where acceptable according to the simulated cut-offs, χ^2^ = 21.799, *CFI* = 0.992, *TLI* = 0.984, *RMSEA* = 0.038, and *SRMR* = 0.025. Standardized factor loadings were greater than or equal to λ = 0.645 for all six indicators and the latent variables correlated at *r* = −0.570. Reliability for both subscales was good, ω_*VBD*_ = 0.853 and ω_*RBI*_ = 0.756.

Next, we tested for measurement invariance in both questionnaire variants (FKB-6_*r*__*eal, ideal*_—see Tables 4a, b) across sex and age groups using the confirmatory and the full sample, respectively. For both variants no meaningful deviation was observed when considering age groups, as evidenced by strict factorial invariance. This indicates that group means resulting from the measurement model can be compared meaningfully. This was also true when analyzing sex for the ideal body image model, but not for the real body image model. Here we observed evidence for metric invariance, but then we found substantial differences in terms of the item intercepts. In particular, items 8 and 10 of the RBI scale evinced standardized intercept differences of Δν = −0.354 and −0.253, respectively. Negative values mean that females score higher than males on this item at the same factor score. In contrast, men scored higher on items 7 and 14 of the VBD scale, as evidenced by positive standardized intercept differences of Δν = 0.291 and 0.256, respectively. By allowing the intercept of item 8 to vary between groups, we established partial strict invariance across sexes (Steenkamp and Baumgartner, 1998).

**TABLE 4a:** Tests of measurement invariance of the FKB-6 real.

**Model**	**χ^2^ (*df*)**	**Δχ^2^**	**Δ*df***	***p***	***CFI***	**Δ*CFI***	***RMSEA***	**Δ*RMSEA***
**Sex**								
Configural invariance	26.352 (16)				0.996		0.037	
Female	21.838 (8)				0.988		0.059	
Male	4.745 (8)				1		0.000	
Metric invariance	27.508 (20)	1.156	4	0.121	0.997	0.001	0.028	0.009
Scalar invariance	57.165 (24)	29.658	4	0.001	0.987	0.010	0.052	0.024
Partial scalar invariance^*a*^	41.841 (23)	14.333	3	0.009	0.993	0.004	0.041	0.013
Partial strict invariance^*a*^	52.178 (29)	10.337	6	0.005	0.989	0.004	0.043	0.002
**Age**								
Configural invariance	80.194 (48)				0.993		0.047	
18–29	13.512 (8)				0.993		0.044	
30–39	13.625 (8)				0.991		0.053	
40–49	12.810 (8)				0.995		0.040	
50–59	12.640 (8)				0.993		0.048	
60–69	15.901 (8)				0.991		0.051	
≥70	11.702 (8)				0.994		0.042	
Metric invariance	105.688 (68)	25.493	20	0.002	0.992	0.001	0.042	0.005
Scalar invariance	136.984 (88)	31.296	20	0.006	0.990	0.002	0.041	0.001
Strict invariance	164.732 (128)	27.748	40	0.015	0.988	0.002	0.038	0.003

*^*a*^The intercept of Item 8 was freed to vary between groups.*

**TABLE 4b:** Tests of measurement invariance of the FKB-6 ideal.

**Model**	**χ^2^ (*df*)**	**Δχ^2^**	**Δ*df***	***p***	***CFI***	**Δ*CFI***	***RMSEA***	**Δ*RMSEA***
**Sex**								
Configural invariance	25.907 (16)				0.994		0.040	
Female	17.694 (8)				0.990		0.052	
Male	9.318 (8)				0.998		0.022	
Metric invariance	27.903 (20)	1.996	4	0.111	0.996	0.002	0.031	0.009
Scalar invariance	34.174 (24)	6.271	4	0.081	0.995	0.001	0.032	0.001
Strict invariance	41.552 (30)	7.377	6	0.078	0.993	0.002	0.032	0.000
**Age**								
Configural invariance	67.325 (48)				0.994		0.040	
18–29	11.170 (8)				0.995		0.037	
30–39	3.683 (8)				1.00		0.000	
40–49	10.346 (8)				0.996		0.034	
50–59	10.736 (8)				0.994		0.044	
60–69	14.981 (8)				0.989		0.052	
≥70	20.438 (8)				0.978		0.078	
Metric invariance	91.364 (68)	24.039	20	0.030	0.993	0.001	0.036	0.004
Scalar invariance	115.355 (88)	23.991	20	0.026	0.993	0.000	0.033	0.003
Strict invariance	138.220 (128)	22.864	40	0.253	0.994	0.001	0.026	0.007

Finally, it could be confirmed that discrepancies between the real and the ideal body image (in VBD and RBI) are positively associated with somatoform (VBD = 0.184; RBI = 0.071) and body dysmorphic symptoms (VBD = 0.141; RBI = 0.142) and negatively with physical and subjective well-being as well as with quality of life. As seen in Table 6, low to moderate correlations were shown.

### Validity Correlations

We correlated the scales of the real and the ideal version of the FKB-6 with other related measures to test convergent validity (see Table 5). Correlations were present as hypothesized. Physical and subjective well-being, as well as quality of life are positively correlated with VBD, but negatively related to RBI. As shown in Table 5 the strongest positive correlation in terms of VBD was with *resilience* subscale of the FEW (*r*_*real*_ = 0.77; *r*_*ideal*_ = 0.56), while the weakest was with somatic symptoms (*r*_*real*_ = −0.53; *r*_*ideal*_ = −0.40). As further hypothesized, somatoform and body dysmorphic symptoms were positive correlated with RBI, but negatively or low with VBD. The highest positive correlation in terms of RBI was with specific symptoms of the BDSI scale (*r*_*real*_ = 0.49; *r_*ideal*_* = 0.38), while the lowest was with health-related quality of life (*r*_*real*_ = −0.41; *r*_*ideal*_ = −0.32).

**TABLE 5:** Associations of the real and ideal version of the FKB-6 with related measures.

	**EURO**	**WHO-5**	**FEW-B**	**FEW-V**	**FEW-G**	**FEW-I**	**FEW_*To*__*tal*_**	**SOMS-7**	**BDSI1**	**BDSI2**	**BDSI_*To*__*tal*_**
VBD_*Real*_	0.668**	0.626**	0.779**	0.649**	0.637**	0.596**	739**	−0.537**	−0.332**	−0.182**	−0.334**
RBI_*Real*_	−0.414**	−0.280**	−0.386**	−0.326**	−0.389**	−0.343**	−0.398**	0.306**	0.499**	0.227**	0.496**
VBD_*Ideal*_	0.524**	0.397**	0.561**	0.447**	0.435**	0.398**	0.512**	−0.404**	−0.233**	−0.124**	−0.234**
RBI_*Ideal*_	−0.328**	−0.199**	−0.304**	−0.255**	−0.310**	0.279**	−0.317**	0.261**	0.388**	0.180**	0.385**

*All correlations were highly significant ^∗∗^(*p* < 0.001). RBI, Rejecting Body Image; VBD, Vital Body Dynamic; EURO, EURO-HIS-QOL; FEW, Physical well-being; FEW-B, Resilience; FEW-V, Vitality; FEW-G, Enjoyment; FEW-I, Inner Peace; BDSI1, Body dimorphic symptoms; BDSI2, specific body parts.*

**TABLE 6:** Correlations between discrepancies of the real vs. ideal and related measures.

	**EURO**	**WHO**	**FEW-B**	**FEW-V**	**FEW-G**	**FEW-I**	**FEW_*To*__*tal*_**	**SOMS**	**FK1**	**FK2**	**FKS_*To*__*tal*_**
VBD_*dis*_	−0.245**	−0.224**	−0.262**	−0.204**	−0.231**	−0.226**	−0.255**	0.184**	0.140**	0.097**	0.141**
RBI_dis_	−0.128**	−0.103**	−0.106**	−0.072**	−0.120**	−0.113**	−0.113**	0.071*	0.140**	0.084*	0.142**

*All correlations were highly significant ^∗∗^(*p* < 0.001). RBI_*dis*_, Discrepancy Rejecting Body Image; VBD_*dis*_, Vital Body Dynamic Discrepancy; EURO, EURO-HIS-QOL; FEW, Physical well-being; FEW-B, Resilience; FEW-V, Vitality; FEW-G, Enjoyment; FEW-I, Inner Peace; BDSI1, Body dimorphic symptoms; BDSI2, specific body parts.*

### Sociodemographic Influences

We tested for differences in the subscales of the real and ideal body image scale with regard to sociodemographic variables (see Table 1). Almost all comparisons were significant, which was expected given the sample size. Age, family status, and education had barely an effect in explaining differences in body image rejection (in both versions). Regardless, the two last variables did have a very small effect, but only in the real body image version. On the other hand, sex and education (real version) exhibited slightly larger effect sizes. The largest effect was, however, traceable to family status (real version) which explained close to 20% of variance.

In terms of vital body dynamics sex (ideal version) had barely an effect in explaining differences. However, in the real version sex exhibited a slightly larger effect size, as well as education (both versions) and family status (ideal version). The largest effect was attributed to age and employment (both versions) which explained more than 10% of the variance.

In sum, compared to other variables family status had the largest effect in explaining differences concerning rejecting body image (real version). With reference to vital body dynamics age and employment were the most influential variables (both versions).

### Norm Values

In Tables 7–7c, we report percentile ranks partitioned by sex and by age groups.

**TABLE 7:** Normative percentile ranks for the VBD real version.

**Sum Score**	**Female**						**Male**					
	**Age in years**						**Age in years**					
	**18–29 (*n* = 215)**	**30–39 (*n* = 239)**	**40–49 (*n* = 219)**	**50–59 (*n* = 186)**	**60–69 (*n* = 207)**	**≥70 (*n* = 162)**	**18–29 (*n* = 215)**	**30–39 (*n* = 196)**	**40–49 (*n* = 201)**	**50–59 (*n* = 162)**	**60–69 (*n* = 227)**	**≥ 70 (*n* = 138)**
3	0	0	1	1	1	5	0	0	1	1	1	5
4	1	1	2	2	2	8	0	1	2	2	2	7
5	2	2	3	4	3	13	0	3	5	4	7	10
6	4	3	5	8	10	23	1	5	7	6	11	17
7	7	6	8	12	14	38	3	7	10	9	18	28
8	12	8	13	18	24	49	3	8	18	15	22	33
9	16	13	17	31	45	64	7	13	24	20	34	50
10	23	21	29	42	56	77	12	19	34	30	48	65
11	30	34	42	59	68	87	15	23	60	46	62	75
12	47	59	71	80	87	96	33	45	76	65	87	92
13	67	80	86	88	95	98	51	63	85	75	94	96
14	80	90	94	95	98	99	67	77	85	86	97	99
15	100	100	100	100	100	100	100	100	100	100	100	100

*VBD, Vital Body Dynamics.*

**TABLE 7a:** Normative percentile ranks for the RBI real version.

**Sum score**	**Female**						**Male**					
	**Age in years**						**Age in years**					
	**18–29 (*n* = 215)**	**30–39 (*n* = 239)**	**40–49 (*n* = 219)**	**50–59 (*n* = 186)**	**60–69 (*n* = 207)**	**≥ 70 (*n* = 162)**	**18–29 (*n* = 215)**	**30–39 (*n* = 196)**	**40–49 (*n* = 201)**	**50–59 (*n* = 162)**	**60–69 (*n* = 227)**	**≥70 (*n* = 138)**
3	29	31	23	23	26	30	48	46	38	41	40	34
4	44	49	44	41	40	48	63	60	54	58	49	49
5	60	66	56	54	52	65	76	72	64	69	61	60
6	71	75	68	63	67	77	83	82	76	77	73	69
7	83	80	79	76	76	86	89	88	85	84	80	78
8	87	87	84	83	86	93	92	91	89	87	90	86
9	92	93	90	90	94	96	93	95	93	92	95	93
10	94	95	92	94	96	98	96	96	95	94	98	93
11	96	97	96	96	99	99	99	98	97	99	99	98
12	97	98	97	98	99	100	99	99	99	100	99	99
13	98	99	99	99	99		99	99	100		100	100
14	99	100	100	100	100		100	99	100		100	
15	100	100		100			100	100	100			

*RBI, Rejecting Body Image.*

**TABLE 7b:** Normative percentile ranks for the VBD ideal.

**Sum score**	**Female**						**Male**					
	**Age in years**						**Age in years**					
	**18–29 (*n* = 215)**	**30–39 (*n* = 239)**	**40–49 (*n* = 219)**	**50–59 (*n* = 186)**	**60–69 (*n* = 207)**	**≥70 (*n* = 162)**	**18–29 (*n* = 215)**	**30–39 (*n* = 196)**	**40–49 (*n* = 201)**	**50–59 (*n* = 162)**	**60–69 (*n* = 227)**	**≥70 (*n* = 138)**
3	0	0	0	0	0	4	0	0	0	2	1	4
4	0	0	0	0	0	6	0	0	0	3	1	7
5	1	1	2	1	2	12	0	0	0	4	4	9
6	3	2	3	4	4	19	0	1	2	6	8	16
7	6	4	5	8	11	30	1	4	4	9	13	20
8	11	6	7	11	20	40	2	7	9	15	21	33
9	18	12	16	23	34	51	8	11	15	24	34	45
10	29	19	23	33	46	64	13	17	25	30	42	56
11	37	36	35	46	57	77	20	25	35	41	54	73
12	56	59	63	69	83	86	35	45	53	63	77	85
13	70	76	76	82	89	92	47	59	67	77	85	92
14	80	87	88	90	94	92	66	74	78	84	92	94
15	100	100	100	100	100	100	100	100	100	100	100	100

*VBD, Vital Body Dynamics.*

**TABLE 7c:** Normative percentile ranks for the RBI ideal.

**Sum score**	**Female**						**Male**					
	**Age in years**						**Age in years**					
	**18–29 (*n* = 215)**	**30–39 (*n* = 239)**	**40–49 (*n* = 219)**	**50–59 (*n* = 186)**	**60–69 (*n* = 207)**	**≥ 70 (*n* = 162)**	**18–29 (*n* = 215)**	**30–39 (*n* = 196)**	**40–49 (*n* = 201)**	**50–59 (*n* = 162)**	**60–69 (*n* = 227)**	**≥70 (*n* = 138)**
3	25	25	23	26	21	28	44	39	33	36	28	30
4	37	39	43	44	33	44	55	55	46	47	42	46
5	46	55	58	54	45	58	63	65	61	61	54	58
6	63	70	70	62	63	73	71	77	72	67	65	72
7	74	78	77	73	78	81	80	83	81	72	75	83
8	84	85	84	81	86	89	86	88	86	80	83	86
9	91	90	92	88	94	94	93	94	92	86	93	92
10	93	93	95	94	97	98	96	97	95	93	96	96
11	96	97	98	98	98	98	98	99	98	96	98	99
12	98	98	99	99	100	100	100	99	100	98	100	100
13	98	99	100	99	100		100	99	100	99	100	
14	99	100		100	100		100	100		100		
15	100				100							

*RBI, Rejecting Body Image.*

## Discussion

The main aim of the present study was to provide a short version of the FKB-20 (real and ideal version) and demonstrate its validity by means of associated constructs. Such a measure would economize clinical assessments and research endeavors. A further aim, was to illustrate the effects related to discrepancies between a real and an ideal body image, in the matter of somatic and body dysmorphic symptoms, quality of life, psychological and physical well-being.

The psychometric analyses evinced satisfactory results for both versions of the scale. The reliability indices were similar to those mentioned in previous studies (Clement and Löwe, 1996; Albani et al., 2006a,b; Daig et al., 2006; Hinz et al., 2010). The overall results suggested that both short versions of the FKB are valid and reliable providing accurate measurement of appraisals, feelings, and dynamics related to body image issues. Further, it was revealed that large discrepancies between a real and an ideal body image are correlated with somatic and body dysmorphic symptoms, leading to chronic stress and reduced well-being, as previously reported (Schmidt et al., 2012). This outcome suggested, that being in discomfort with one’s ideal body image and in struggle to accept the real body image leads to body dissatisfaction has negative consequences. In support of this, past evidence associates body image discrepancies (ideal vs. real) with a range of negative health outcomes such as depression and hypochondriasis (e.g., Cash and Smolak, 2011; Wilson et al., 2013; França et al., 2017; Becker et al., 2019). In addition, it seems plausible to assume that discrepancies between the real and the ideal body image, may lead to a state of cognitive dissonance, promoting internal discomfort and stress (Festinger, 1957; Dilakshini and Kumar, 2020). These fits comparable findings in previous studies (Benninghoven et al., 2007; Hrabosky and Grilo, 2007) confirming the growing evidence of body dissatisfaction (Halliwell et al., 2011; Wang et al., 2019) as a result of incongruences between beauty ideals and current body sizes. Some researchers even emphasized that ideal comparisons strongly affected body image, especially in women (Betz et al., 2019).

Strict measurement invariance across age and sex was found for the ideal version. This was also true for the real version when considering age, but not for sex. However, partial invariance was established in this case. This is a relevant and novel finding regarding the measurement of body image, since this reveals that the measurement model is identical for these groups. If these prerequisites were not fulfilled, comparisons of latent and observed means and variances between groups would be questionable (Gregorich, 2006; Schmalbach and Zenger, 2019). Specifically, between-group equivalence of factor loadings and item intercepts is needed for comparisons of latent and observed means. On the other hand, merely the equivalence of factor loadings is needed to allow for comparisons between latent variances, but equivalence of both, loadings and item residual variances is required for meaningful comparisons of observed variances. We found small between-group intercept differences for several items when considering the real body image questionnaire.

The largest intercept deviation was exhibited for Item 8 (“Unhappy with one’s figure”).

This makes sense since compared to men, women are generally more unhappy with their body, even across age (Quittkat et al., 2019). Even though men are also affected (Frederick et al., 2012), it has been concluded that body dissatisfaction tends to be higher in women than in men (Betz et al., 2019; Quittkat et al., 2019; Radwan et al., 2019). Possible explanations are the particular propensity of women to “misperceive” their body weight (Chang and Christakis, 2001) and the higher relevance of appearance in women than in men (Quittkat et al., 2019), which recurrently manifests in an incremented eating disorder prevalence in women than in men (Galmiche et al., 2019).

Scalar invariance was then only obtained after relaxing the equality constraint for one item (Item 8), This means that—even given the same factor score—one will still find differences in the observed means between groups. As pointed out by Steinmetz (2013), observed means should thus not be taken at face value for the Rejecting Body Image subscale of the real body image questionnaire. Instead, researchers should examine latent means if they are interested in differences between sexes.

Evidence of validity was exhibited by means of correlations of the FKB-6 (both versions) with other related constructs. As expected, convergent validity was indicated by means of strong correlations between vital body dynamics and physical well-being, quality of life, and subjective well-being. This shows that VBD is well reflected in these qualities. The strongest correlation was shown between VBD and physical well-being properly emphasizing the physical component in this dimension. Previous studies demonstrated similar results, underlining the relevance of these findings (Clement and Löwe, 1996; Albani et al., 2006a,b, 2009). On the other hand, somatoform and body dysmorphic symptoms provide evidence of divergent validity, since they are negatively related to VBD. The weakest correlation was observed with somatoform symptoms. Consequently, VBD clearly distinguishes itself from body centered disturbances and complaints. Similar results were shown in previous research (Sack et al., 2002; Albani et al., 2006a, 2009). Convergent and divergent validity for the rejecting body image was also evident. Moderate to strong correlations with somatoform and body dysmorphic symptoms indicate convergent validity. The strongest correlation is observed with body dysmorphic symptoms. As a result, this highlights the aspect of body discomfort and preoccupation underlined in rejecting body image. Some researchers have reported comparable findings even among athletes (Daig et al., 2006, 2008; Dyl et al., 2006; Rief et al., 2006; Albani et al., 2009; Sarrar et al., 2010). Further, it was observed that the correlations in the ideal version, as expected, are similar to the ones in the real version, however, they are slightly smaller. An explanation for this, is that the ideal version of the FKB-6 is being compared to total scores (of the other scales) reflecting real and not *ideal* values of the participants.

Comparisons between sociodemographic variables revealed that in both versions age and employment were the most influential variables in terms of explaining differences in vital body dynamics. On the other hand, family status had the largest effect regarding rejecting body image (real version). This goes in line with literature on body image, showing changes in body image perception relevant to age, employment and family status (Grogan, 1999, 2017; Paeratakul et al., 2002; Albani et al., 2006a; Myers and Crowther, 2009; Klos and Sobal, 2013).

### Limitations

The present study utilized primarily a statistical approach grounded in classical test theory (CTT). A growing body of research has discussed differences between CTT and an alternative approach: Item response theory (IRT; Embretson and Reise, 2013). In most cases, the two approaches lead to similar results (Fan, 1998; Kamata and Bauer, 2008; Progar et al., 2008; Sébille et al., 2010). Nonetheless, we acknowledge that our focus on CTT over IRT paints a potentially incomplete picture of the scale construction process. Thus, future validation studies could benefit from implementing the IRT. In addition, the validation of the present scale is based on non-clinical data. We included and analyzed data of the general population and provided norm values, which is useful for the evaluation of clinical samples. Notwithstanding, following studies could enrich the validity of the scale by focusing on providing psychometric properties based on clinical samples.

## Conclusion

In the face of growing body image disturbances and its pervasive effects on physical and mental health, there is a crucial demand in the field of body image assessment to provide proper measures, especially given the need of such in German-speaking populations. The FKB-6 aims to meet these demands providing an economic and a validated tool that is best of use in in research settings and economic clinical assessments. The short versions of the FKB-20 are valid and reliable instruments that measure body image issues by means of affective and cognitive variables. They facilitate and economize diagnosis and aids treatment in clinical contexts. Its brevity provides advantages for large scientific surveys.

## Data Availability Statement

The raw data supporting the conclusions of this article will be made available by the authors, without undue reservation, to any qualified researcher.

## Ethics Statement

Ethical review and approval was not required for the study on human participants in accordance with the local legislation and institutional requirements. The patients/participants provided their written informed consent to participate in this study. The methods of data collection were in accordance with the Helsinki Declaration of 1975 (as revised in 1983).

## Author Contributions

IS contributed to the writing of the draft of the manuscript and managing information of new editions and corrections. CA was responsible for data collection and conception data management. EB contributed to the design of the study idea. BS contributed to the data analysis and discussion. KP and HB contributed to the writing and editing of the manuscript and literature review. MZ contributed to the writing and editing of the manuscript. All authors have contributed to the preparation of the manuscript and report of results, and approve the submitted manuscript for publication.

## Conflict of Interest

The authors declare that the research was conducted in the absence of any commercial or financial relationships that could be construed as a potential conflict of interest.
